# Dietary and Food Consumption Patterns and Their Associated Factors in the Tibetan Plateau Population: Results from 73 Counties with Agriculture and Animal Husbandry in Tibet, China

**DOI:** 10.3390/nu14091955

**Published:** 2022-05-07

**Authors:** Chang Kong, Linsheng Yang, Hongqiang Gong, Li Wang, Hairong Li, Yonghua Li, Binggan Wei, Cangjue Nima, Yangzong Deji, Shengcheng Zhao, Min Guo, Lijuan Gu, Jiangping Yu, Zongji Gesang, Rujun Li

**Affiliations:** 1 Key Laboratory of Land Surface Pattern and Simulation, Institute of Geographical Sciences and Natural Resources Research, Chinese Academy of Sciences, Beijing 100101, China; kongc.15s@igsnrr.ac.cn (C.K.); yangls@igsnrr.ac.cn (L.Y.); wangli@igsnrr.ac.cn (L.W.); yhli@igsnrr.ac.cn (Y.L.); weibg@igsnrr.ac.cn (B.W.); gulj@igsnrr.ac.cn (L.G.); yujp@igsnrr.ac.cn (J.Y.); 2 College of Resources and Environment, University of Chinese Academy of Sciences, Beijing 100049, China; 3 Tibet Center of Disease Control and Prevention, Lhasa 850030, China; rockyghq@126.com (H.G.); nc916@126.com (C.N.); ypmd080406@163.com (Y.D.); zhaoshengcheng_520@163.com (S.Z.); g7275@126.com (M.G.); zongji168@126.com (Z.G.); lirujun19910716@126.com (R.L.)

**Keywords:** dietary pattern, food consumption pattern, food consumption score, Tibetan Plateau, China

## Abstract

Dietary imbalances are an important cause of morbidity and mortality, both in China and globally. Abnormal element content in the natural environment and the unbalanced dietary structure of populations coexist in the Tibetan Plateau. This study analyzed the dietary and food consumption patterns of 617 Tibetan residents and their associated factors. Cluster analysis revealed three modes of dietary pattern; the food consumption scores (FCSs) of subjects in modes with relatively high consumption frequency of staple food and relatively singular dietary structure were the lowest. Although the FCSs of most subjects were acceptable (FCS > 35), subjects with relatively low FCSs were more dependent on locally cultivated highland barley that is probably low in selenium. Hierarchical linear models revealed both individual–family and regional factors were significantly related (*p* values < 0.05) with the food consumption of subjects as follows: age, travel time from township to county, and cultivation area of highland barley were negatively related; numbers of individuals aged 40–60 years and pork, beef, and mutton production were positively related. Individuals with secondary or higher education had higher FCSs. A single indicator may be incomprehensive in dietary and food consumption studies. For people with a relatively unbalanced diet, an analysis of the main foods they consume is critical. Dietary and food consumption patterns might have relatively large inter-regional and intra-regional variations; therefore, factors that influence it might be multi-level and multi-scale.

## 1. Introduction

Balanced dietary patterns are an important cornerstone for the prevention of related chronic diseases; however, worldwide, the absolute disease burden caused by dietary risks has risen in the last 30 years [1]. Of the unbalanced diets, low fruit and whole grain consumptions were among the top 10 risk factors for death and disability-adjusted life years globally and in many countries [1,2]. In China, dietary imbalances are a leading cause of morbidity and mortality [3]. 

Environmental, social, economic, and individual factors influence dietary structure. Ecological factors include seasonal variations, land-use diversity, climate, and the environment; social factors include those related to an aging population, urbanization, cultural diversity, and festivals; and economic factors include income, development, urban–rural economic differences, price, and markets [4]. In addition, it was reported that national income level, personal social status, affordability, health concepts, purchase preference, perceived value, and genetic characteristics could influence dietary structures [5,6,7,8,9,10].

The dietary structure of the people of China is constantly changing with the continuous growth in income and rapid urbanization [11,12,13]. The factors influencing dietary structure are also changing [11,14]. In addition to the changing dietary structure, with increasing carbon dioxide emissions and the resultant global climate change, nutrient content and crop yields are predicted to decrease, affecting food security [15,16,17,18]. Household food security among smallholder farmers is sensitive to variable and changing climates [19], and in some rural and underdeveloped districts, particularly those in remote and high-altitude localities, the impact of climate change on food consumption safety needs close attention. To counteract and prevent the adverse effects of possible nutrient deficiency or a declining trend in local crops, adjustments in dietary structure could be an effective way to balance nutrient levels in the human body.

Tibet is located on the Tibetan Plateau and was once a Kashin–Beck disease-endemic area, which might be closely related to its biogeochemical selenium deficiency [20,21,22]. Through government efforts and medical and research departments, Kashin–Beck disease was stably controlled by exchanging grain and relocation. Nevertheless, a low-selenium environment still exists, and thus, special dietary structure needs consideration. 

The dietary structure of Tibetan residents is relatively singular, and the intake of foods such as fruits and vegetables is insufficient [23,24]. This is one of the important causes for the high incidence of cardiovascular and cerebrovascular diseases in Tibet. Although the dietary structure in Tibet has changed significantly in recent years, there are still regional differences [25,26].

Although there have been some studies on food consumption and dietary structure of the Tibetan Plateau residents [27,28,29], these studies selected representative regions or populations for analysis, and few field investigations throughout rural Tibetan areas have been conducted. Therefore, it is difficult to reflect internal differences such as regional or individual differences. Based on food frequency questionnaires, this study aimed to (1) analyze the main dietary patterns, food consumption scores (FCSs), and characteristics of staple food consumption and their regional differences in the Tibetan Plateau population; (2) discuss the dietary and food consumption patterns through analyses combing three indicators; (3) model food consumption and its influencing factors.

## 2. Materials and Methods

### 2.1. Study Area and Investigation

**Figure 1:**
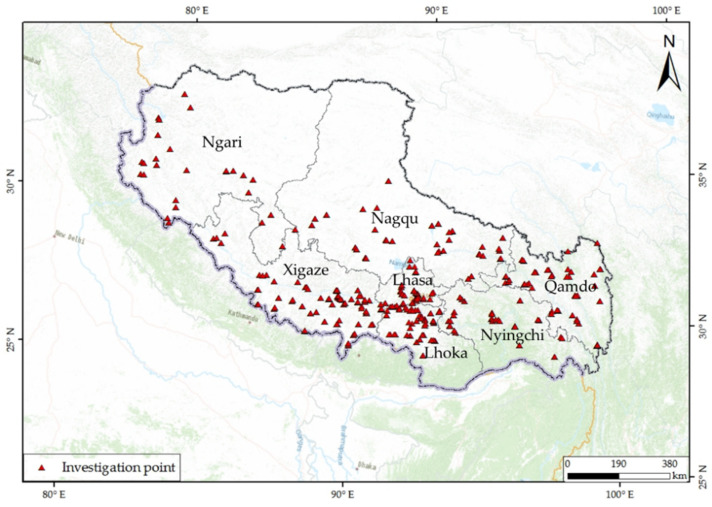
Location of residents who participated in the investigation in Tibet, China.

The study protocol was approved by the Tibet Center of Disease Control and Prevention and the municipal- and county-level center of disease control and prevention. Procedures in this study were in accordance with the ethical standards of the Institutional Research Committee and with the 1964 Helsinki Declaration and its later amendments, or comparable ethical standards. All individuals were informed in advance and consented to participate this survey. In 2019–2021, questionnaire surveys were conducted in all 73 counties of agriculture and animal husbandry, except for Chengguan District in Tibet (Figure 1). The stratified sampling method was used to select two townships from each county, two villages from each township, and two households from each village. The principle of sampling township/village sites being as spatially dispersed as possible was followed, and a random sample of permanent households was taken for the investigation. Sample size was adjusted during the investigation, and finally, 617 questionnaires were obtained. 

The questionnaire comprised 23 questions in three sections, including information on (1) individual factors (such as gender, age, and education attainment); (2) family characteristics (such as number of family members and their age and family annual income); (3) the consumption frequency of 13 kinds of foods in the previous month. According to local dietary habits, the types of food investigated in this study were rice, meat, poultry, eggs, fruits, vegetables, and oil, including the following foods and their products: highland barley, wheat, corn, potato, milk, and beans. Salt is an essential part of the Chinese diet, and fish consumption by Tibetan residents was very low according to our field investigation; therefore, these were not included in this study. Considering that the food consumption structure of Tibetan residents is relatively singular, and some foods are consumed monthly, the consumption frequency was investigated using a calendar month as the unit before being converted into a weekly frequency.

### 2.2. Calculation Method of FCS

The FCS is an indicator proposed by the United Nations World Food Program. It is a comprehensive score based on dietary diversity, food consumption frequency, and the nutritional importance of food groups. This food consumption indicator is able to capture dietary diversity and food frequency and also reflects the level of family food security. Based on the typical thresholds [30], household food security profiles were constructed as poor (FCS < 21), borderline (FCS range of 21.5–35), and acceptable (FCS > 35).

The Technical Guide Sheet by the World Food Programme [30] describes noting the nutritional importance of food groups as the weight (Table 1). The FCSs were calculated by adding the multiplication of the corresponding standard weights of the weekly consumption frequency of food groups. The formula is shown below as Formula (1).

(1) $$\mathrm{FCS}=\;\sum_{i=1}^{i=7}frequency_{i}\times weight_{i}$$

### 2.3. Analysis on Influencing Factors of Food Consumption

The results of previous studies have shown that individual and family factors were perceived as important factors of food consumption [4,20,31], so individual and family factors such as age, gender, educational attainment, number and age of family members, and family annual income were included in this study.

During the field investigation, there are regional differences in the diversity of foods available for purchase. Consequently, in this study, in addition to personal and family factors, county attributes were also considered. Environmental, social, and economic factors influence dietary structure [4,7,8], so we selected factors from these aspects based on local conditions. Tibetan residents are relatively dependent on local food, so we selected planting and breeding conditions and output values of the agriculture, forestry, animal husbandry and fishery service industries. Considering the availability as well as the affordability of food for residents of different counties, population sizes, administrative areas, traffic conditions, and economic levels of counties were selected. In all, 30 variables were explored; these potential confounding influencing factors of FCS were divided into two levels: individual–family- and county-level influencing factors (Table 2).

Individual and family information was extracted from the questionnaire, and socioeconomic information of regions was collected from the Tibet statistical yearbook 2020 [32] and the China statistical yearbook (county level) 2020 [33]. Town road data used to calculate the travel time from township to county were sourced from OpenStreetMap (https://www.openhistoricalmap.org (accessed on 14 January 2021)) for 2020 and the Resources and Environment Sciences and Data Center of Chinese Academy of Sciences (http://www.resdc.cn/lds.aspx (accessed on 19 January 2021)).

**Table 2:** Classification and description of potential confounding factors related to food consumption score for the study analysis.

Independent Variable	Classification and Description
**Individual–family-level factors**	
Age	Continuous variables
Gender	1 = Male; 2 = Female
Educational attainment	1 = No education; 2 = Primary; 3 = Secondary or higher
Number of family members	Continuous variables
Number of family members under 18 years of age	Continuous variables
Number of family members aged 18–40 years	Continuous variables
Number of family members aged 40–60 years	Continuous variables
Number of family members over 60 years of age	Continuous variables
Family annual income (CNY 10,000)	Continuous variables
**County level factor**	
Counties by type	1 = county of agriculture; 2 = county of half agriculture and half animal husbandry; 3 = county of animal husbandry
The travel time from township to county (minute)	Continuous variable ^a^
Population	Continuous variable
Administrative area (square kilometer)	Continuous variable
Population density (person per square kilometer)	Continuous variable ^b^
Regional gross domestic product (CNY 10,000)	Continuous variable
Planting area of facility agriculture (hectare)	Continuous variable
Cultivation area of food crop (hectare)	Continuous variable
Cultivation area of wheat (hectare)	Continuous variable
Cultivation area of highland barley (hectare)	Continuous variable
Agricultural acreage at the end of year (hectare)	Continuous variable
Number of livestock stocks at the end of the year (10,000)	Continuous variable
Number of heavy livestock stocks (10,000)	Continuous variable
Pork, beef, and mutton production (ton)	Continuous variable
Output value of agriculture, forestry, animal husbandry and fishery (CNY 10,000)	Continuous variable
Output value of farming (CNY 10,000)	Continuous variable
Output value of forestry (CNY 10,000)	Continuous variable
Output value of animal husbandry (CNY 10,000)	Continuous variable
Output value of fishery (CNY 10,000)	Continuous variable
Output value of agriculture, forestry, animal husbandry and fishery service industry (CNY 10,000)	Continuous variable
Gross output value of industry (CNY 10,000)	Continuous variable

Notes: ^a^, the travel time from township to county was used to characterize the accessibility of residents to market food, with reference to the method used by Huang [34]; ^b^, population density was calculated by dividing population by administrative area.

The subjects and counties were nested; therefore, this study used hierarchical linear models (HLMs) by stage modeling to analyze the influencing factors of FCS. According to the geographical distribution of food consumption patterns, food consumption varied inter- and intra-regionally. 

To explain individual differences in generalization, we introduced a random intercept HLM. This model is given by Formula (2) to (4).

Level 1:

(2) $$Y_{ij}=\beta_{oj}+\sum_{k=1}^{K}\beta_{kj}X_{kij}+\varepsilon_{ij},\;\varepsilon_{ij}\sim N\left(\sigma_{\varepsilon }^{2}\right)$$

Level 2:

(3) $$\beta_{0j}=\gamma_{00}+\sum_{m=1}^{M}\gamma_{0m}W_{mj}+u_{0j},\;u_{0j}\sim N\left(\sigma_{\beta }^{2}\right)$$

(4) $$\beta_{kj}\;=\gamma_{k0}\;$$

where *i* represents subjects; *j* represents counties; *X_kij_* and *W_mj_* represent independent variables in level 1 and 2; *Y_ij_* represents outcome variables (FCS in this study) for the *i*th subject living in *j*th county; *β*_0*j*_ represents the intercept for *j*th county; *γ*_00_ represents the average intercepts across the counties; *β_kj_* is the slope for the *k*th predictor for county *j*, consisting of a fixed part, *γ_k_*_0_; *γ*_0*m*_ is the slope for the *m*th predictor; and *ε_ij_* and *u*_0*j*_ denote the subject- and county-level residuals, respectively.

A null model was used to evaluate the significance of the differences in FCSs in different counties. In stage 1 of the model, the individual–family factors were entered into the model to examine their strength of association with the FCS, followed by a manual stepwise backward elimination process at a 5% significance level. In stage 2 of the model, the county-level factors were added to the retained significant variables in stage 1 and again, those significant variables were retained. The goodness of fit for each model was determined from Akaike’s information criterion (AIC) of each model, where smaller values indicate better models [35].

### 2.4. Statistical Analysis

The spatial distribution of dietary and food consumption indicators was drawn in ArcMap (version 10.7, ESRI Inc., Redlands, CA, USA). Cluster analyses to determine the classification of food consumption in SPSS (version 19.0, IBM Corp, Armonk, NY, USA) and the food consumption models were conducted in STATA (version 16.0, StataCorp LP, College Station, TX, USA). The result of the cluster analysis was verified using one-way ANOVA to test the significance of variables between the clusters. The significance of the HLM model was verified using the chi-square test. We considered *p* values < 0.05 (two-tailed) as statistically significant.

## 3. Results

### 3.1. Characteristics of the Subjects

**Figure 2:**
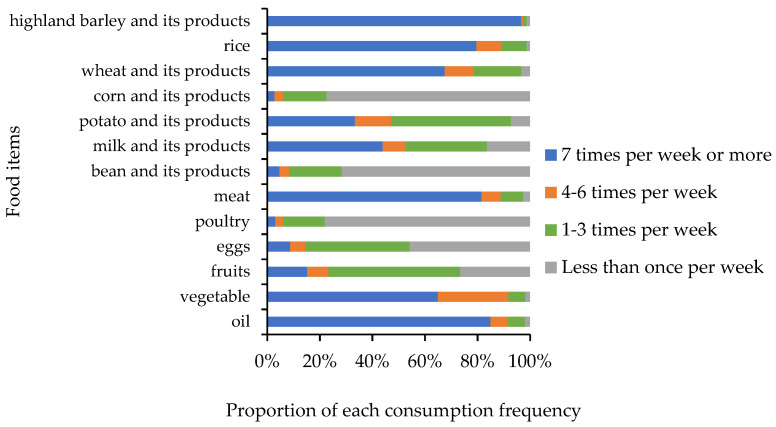
Food consumption frequency of the subjects (*n* = 617).

The final sample consisted of 617 subjects with a mean age of 50 (standard deviation = 13.69). A total of 44.55% of the subjects were males. Nearly half of the subjects (49.73%) had not received an education, and 37.34% of the subjects had received primary education. The consumption frequency of highland barley and its products, rice, wheat and its products, meat, vegetables, and oil was relatively high (Figure 2), while the standard deviation of vegetable consumption frequency was the second largest after milk and its products among these 13 kinds of food. The consumption frequency of vegetables varied considerably among the subjects. 

### 3.2. Dietary and Food Consumption Patterns 

#### 3.2.1. Classification of Dietary Patterns

The cluster analysis of various food consumption frequencies shows that the dietary patterns of subjects can be divided into three modes. In the first mode, the consumption frequency of staple food, fruits, and vegetables was relatively high, and the structure was diversified; the second mode had a relatively high consumption frequency of staple food, meat, and milk, and the structure was less complicated than mode 1; the third mode had a relatively high consumption frequency of staple food, and the dietary structure was relatively singular.

**Figure 3:**
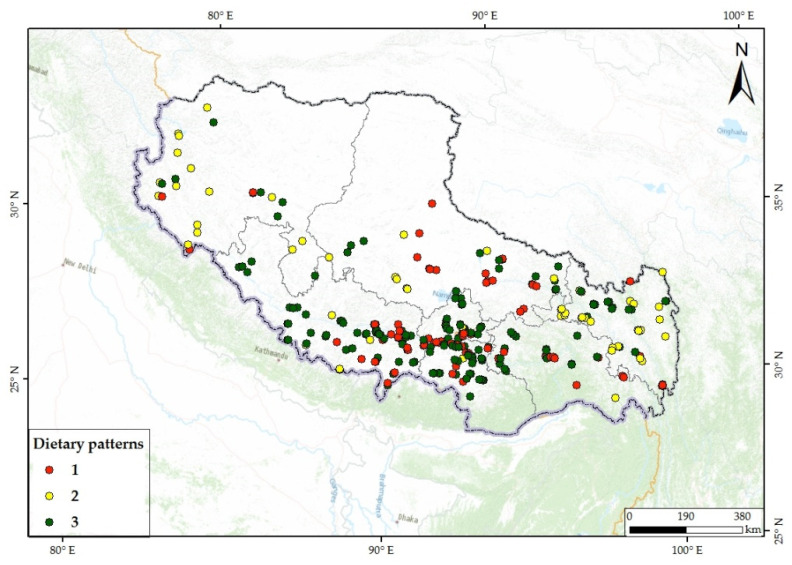
Dietary patterns of subjects in each investigation point. Mode 1: staple food, fruits, and vegetables; Mode 2: staple food, meat, and milk; Mode 3: staple food.

**Figure 4:**
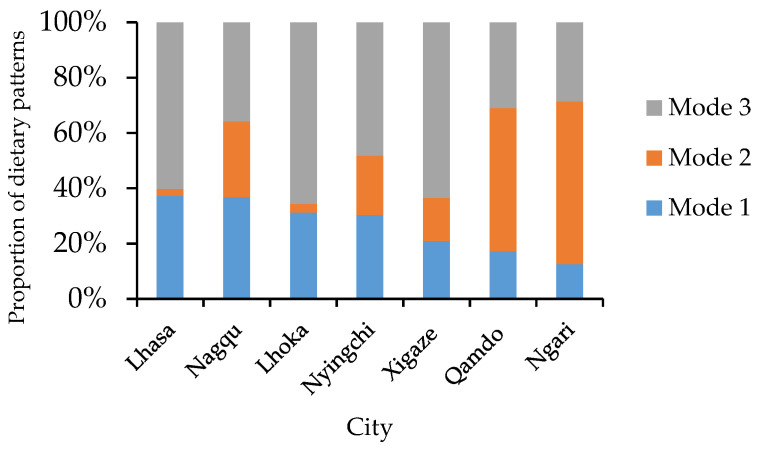
Proportion of dietary patterns in cities.

The proportions of the three modes were 26.74%, 23.34%, and 49.92%, respectively, implying that approximately half of the subjects had comparatively unbalanced dietary patterns. In terms of geographical distribution, the dietary patterns in eastern and central Tibet were more ideal than that in the western regions (Figure 3 and Figure 4). There were observed differences in the composition of the three modes among cities (Figure 4).

#### 3.2.2. FCS 

**Figure 5:**
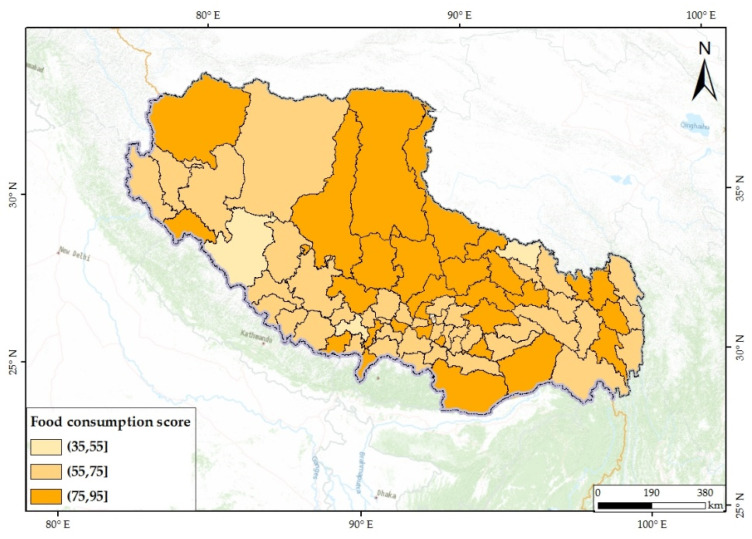
Mean value of food consumption score of subjects in counties.

**Figure 6:**
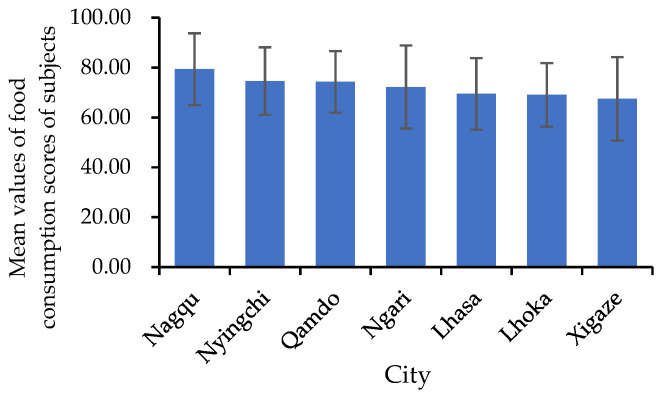
Mean values of food consumption scores of subjects in cities.

The results showed that most of the subjects achieved food security (FCS > 35) [30]. The distribution of the mean value of FCSs showed aggregation in different ranges, and counties with low FCSs were scattered (Figure 5). This suggested that the reasons for low FCSs might be divergent. There were significant differences in FCSs among cities and counties (Figure 6) that might be due to the differences in socioeconomic, natural, and transportation conditions in different regions. 

#### 3.2.3. Proportion of Staple Food Consumption Frequency

Tibetan residents often consume highland barley as an important staple food. However, selenium content in the self-produced main grain highland barley is generally lower than that in purchased grains such as rice and flour [36]. This might be the important cause of low selenium levels in the body and the prevalence of Kashin–Beck disease. Therefore, the proportion of the consumption of various staple foods of Tibetan residents was studied, as well as the regional differences in the proportion of highland barley intake in staple food.

**Figure 7:**
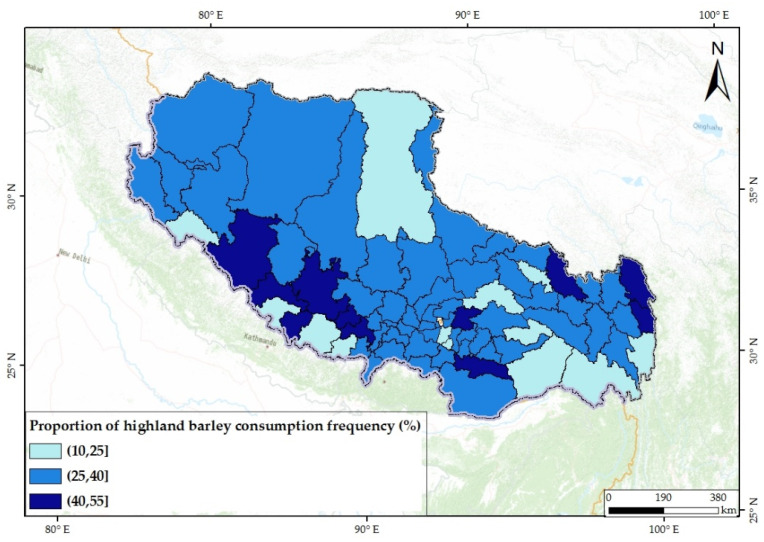
Mean values of the proportion of highland barley consumption frequency of counties.

**Figure 8:**
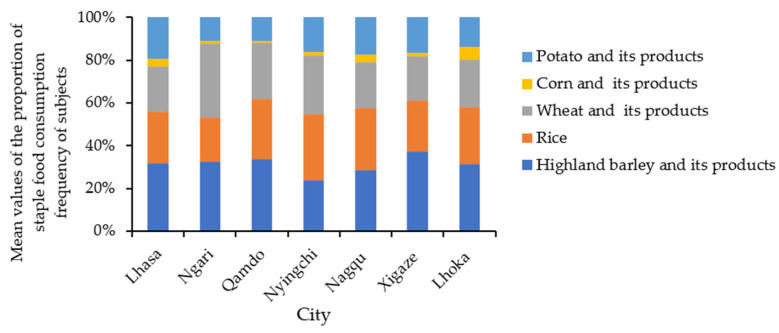
Mean values of the proportion of staple food consumption frequency of subjects in cities in Tibet.

Highland barley accounted for 23.72–37.44% of the staple food consumption frequency at the city level (rice 20.68–31.15%, wheat 20.81–34.51%, potato 11.01–19.29%, and corn 0.80–5.79%). Highland barley, rice, and wheat accounted for approximately 80% of the consumption frequency of staple food, with highland barley remaining the most important staple food for the subjects. The proportion of highland barley in the east and southwest was relatively high, and the dependence on self-produced crops was strong (Figure 7). High-dependent and low-dependent regions were both relatively dispersed. There was variability in the structure of staple food consumption among counties and cities (Figure 8). It is worth noting that the proportions of highland barley and rice were similar, and in some cities and counties, the proportion of rice was higher than that of highland barley. This trend played a positive role in reducing the dependence of residents on local low-selenium crops and increasing the intake level of selenium.

#### 3.2.4. Relationship of Dietary and Food Consumption Indicators

##### Dietary Patterns and FCS

**Figure 9:**
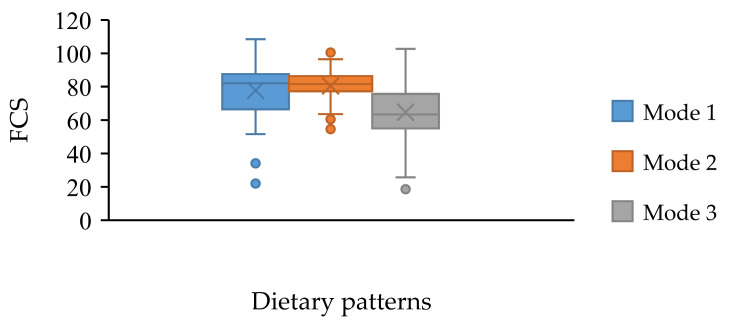
Relationship of dietary patterns and food consumption scores.

After non-parametric testing, the FCSs of the three modes were significantly different (*p* value < 0.01). After pairwise comparison, the FCSs of mode 3 were significantly lower than that of modes 1 and 2 (Figure 9). This demonstrated that subjects who were more dependent on staple food tended to have lower FCSs.

##### Proportion of Staple Food Consumption Frequency and FCS

**Figure 10:**
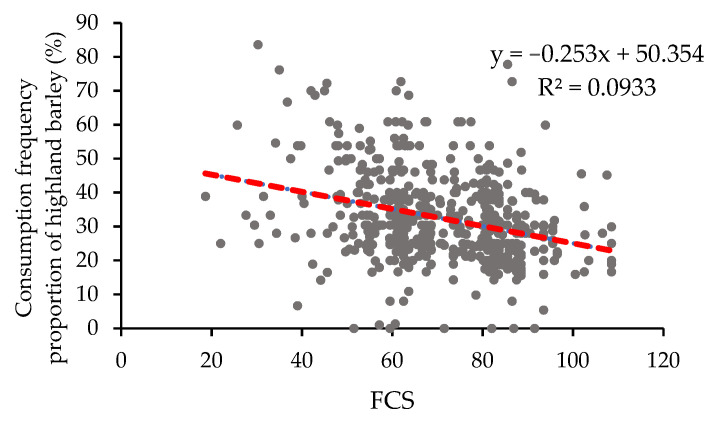
Relationship of the proportion of highland barley consumption frequency (%) and food consumption scores (FCS).

Comparing Figure 5 and Figure 7, it was found that the proportions of highland barley were high in areas with low FCSs. The Pearson’s correlation test showed a negative correlation between these two indicators (Figure 10). This indicates that subjects with relatively low dietary diversity were often more dependent on local low-selenium crops, which might lead to a risk of selenium deficiency in the body.

### 3.3. Factors Associated with Food Consumption

By constructing the null model, the FCSs of counties were found to be significantly different; therefore, the HLM could be constructed in this study. To meet the model requirements of data volume, only groups with a sample size greater than five were retained for further analysis. Questionnaires with missing data were removed. The basic information of the remaining 506 subjects is shown in Table 3. The numbers of males and females were similar, whereas the ages ranged from 19 to 83 years, with the mean value being 49. Over 50% of subjects had not received an education, and subjects that had received secondary education or higher only accounted for 12.25% of the total subjects. The majority of subjects had a household size of less than 10 individuals, and more than half had a household size less than 6. A Pearson’s correlation analysis indicated that each correlation coefficient between the variables was lower than 0.6, indicating good discriminant validity.

Random intercept models were used for the main analyses, and the counties were used as random intercepts. Table 4 provides the detailed findings of all the model analyses of the FCS. All variables in Table 2 were included in the model, and variables not shown in Table 4 had no significant effect on the FCS, as determined by statistical tests; therefore, these factors were eliminated from the models to avoid interference. In model 1, the influence of age on the FCS was not significant; however, it was significant in the single-factor analysis. Consequently, it was entered in model 2. At the individual level, those that were younger and those that had received a secondary or higher education had a significantly higher FCS compared with other ages or educational attainments. Family members aged 40–60 years would contribute to the improvement of the FCS. On the regional scale, the FCS was inversely associated with the travel time from township to county, implying that the residents in townships with good traffic conditions or those residing close to counties would have significantly higher FCSs. Planting and breeding situations also exerted a significant influence on the FCS: the lower the cultivation area of highland barley and the higher pork, beef, and mutton production of the county, the FCSs of the residents were more likely to be higher. 

The travel time from township to county, planting and breeding structure, and traffic conditions had significant impacts on food consumption with small correlation coefficients. The ranges of these three factors were 0–614.56 min, 0–8266.6 hectares, and 352.5–18,962.5 tons, separately, in our analysis; therefore, the maximum differences in FCSs caused by these three factors were found to be 10.01, 14.04, and 13.34, respectively, by multiplying the correlation coefficient and the range of factors. This indicates that although the correlation coefficients were small, these factors exerted an impact on the FCS of more than 10 points. 

This study also analyzed the interaction of influencing factors. Among them, the interaction of the number of family members and family annual income was significant in the unitary analysis but not when they were included in multiple models. The interaction of other influencing factors was not significant. The AICs decreased in the order of the null model, mode 1 and mode 2.

## 4. Discussion

According to this study, there was a large number of subjects whose dietary structure were unitary comparatively, which was consistent with the results of a previous study [3] that showed dietary structures in rural areas need to be improved. In addition, the geographical distribution of dietary patterns, FCS, and proportion of highland barely consumption frequency showed that regional differences in dietary and food consumption patterns still remained. The differences in dietary and food consumption patterns among regions were also found in previous studies in Tibet and other areas [4,7,8,27]. With the development of society, economy, transportation, and agriculture, the availability of various food types and sources has improved. The dependence of dietary structure on local food might become smaller, and regional differences will be narrowed in the future. Therefore, as the availability of diverse foods is gradually improved, the establishment of correct dietary concepts becomes more important.

This study found that the FCSs of most subjects were greater than 35, implying acceptable food security [30]. The FCS was consistent with dietary patterns, and subjects with relatively singular dietary structures had lower FCSs. In addition, by analyzing the relationship of the FCS and the proportion of highland barley consumption frequency in staple food, subjects with lower FCSs showed greater dependence on highland barley. However, in contrast to other staple food, the selenium content in most Tibetan highland barley was relatively low, caused by a large area of selenium-deficient soils in Tibet, especially in Kashin–Beck disease-endemic areas [37,38]. Although the FCS was acceptable, strong dependence on highland barley according to this study might lead to a risk of selenium deficiency if the subjects consume selenium-deficient highland barley. In contrast, highland barley was characterized by superior nutritional functions among grains, and Tibetan highland barley was relatively high in nutritional elements such as calcium, potassium, and sodium [39,40]. Due to the unitary dietary structure, the reduction in highland barley consumption would lead to an increase in the proportion of other staple foods. In 1999, the main staple food of Tibetan people was highland barley and wheat, and highland barley accounted for 70–90% [41]. After over 20 years, the proportion of highland barley and rice was similar. Whether the change in the staple food structure for the Tibetan population has led or would lead to undiscovered or long-term health effects still need to be considered. Therefore, it is necessary to combine indicators reflecting the diversity of food consumption such as the FCS with other indicators based on local food habits and nutrient levels.

As proposed by previous studies [4,27,31], food consumption was affected by both individual–family- and county-level influencing factors in this study. Comparing the results of related studies, food consumption and its significant or leading influencing factors varied across countries or regions [4,8,42,43,44,45]; therefore, it should be analyzed according to population and regional characteristics or other typical characteristics. This study proved that both inter-regional and intra-regional difference in food consumption existed in Tibet. Therefore, the influencing factors were also at different levels. Tibetan food consumption was affected by age, education, family member composition, the travel time from township to county, cultivation area of highland barley, as well as pork, beef, and mutton production. This indicates that planting and breeding structure were still important factors influencing food consumption in Tibet. Furthermore, local traffic conditions and the cognition of individuals and family members also played an important role. Due to insufficient food self-sufficiency, local traffic conditions had impacts on food accessibility, which has been proved to be an influential factor in food consumption [7]. Wang et al. [27] and Zhou et al. [28] found that the elderly retain more traditional Tibetan dietary habits. Similarly, in this study, in agricultural and pastoral areas of Tibet, due to the influence of traditional culture and restrictions of traffic and economic conditions, older residents retained traditional food consumption habits in which the diversity of food consumed was low. Younger residents might have greater possibilities and more diversified ways of accessing various information; therefore, their dietary and living habits might be different. In terms of education factors, it has been shown that a higher level of education improves people’s diets [46]. Meanwhile, allopatric and centralized schooling might be helpful to establish a healthier concept of food consumption. Therefore, there are differences in dietary structure at different ages and educational attainments in the same region, indicating that in addition to local planting and breeding structure and the availability of external food, the dietary concept of residents was also important.

Food consumption and environment are mutually influential. Studies have shown that climate change and air quality changes will affect the nutrient content and the yield of crops [15,16,17,18], which may lead to hunger or hidden hunger. Faced with this situation, a single dietary structure highly dependent on staple food is inadvisable, and a balanced dietary pattern is particularly important to ensure the balance of elements in the human body. At the same time, food consumption can also affect the environment and thus human health [12,47,48,49]. Therefore, the dietary nutrition level of the residents should be further improved, and nutrition monitoring of the population in vulnerable areas still needs to be strengthened to prevent the recurrence of hidden hunger, endemic diseases, and other health problems.

The strengths of our study include combining FCSs, dietary patterns, and the proportion of various staple food consumption frequencies; revealing the geographical characteristics of dietary and food consumption patterns; conducting extensive research on highland farmers and herders; and including related influencing factors of food consumption in different levels. This study provides practical information and guidance on dietary intervention and a new perspective for future studies using dietary and food consumption indicators. However, our study also has a few limitations. First, although we adjusted the model for as many variables as possible, the possibility of missing other confounders could not be ruled out. Second, the effect of natural environmental factors on food consumption was not addressed. Third, the sample size of the surveyed population was relatively small in every county; therefore, the conclusions cannot be generalized for the whole population. Finally, research on the integration of nutrient elements in food and food consumption needs further advancement.

## 5. Conclusions

This study reported that most subjects had achieved acceptable food security; however, they might still have relatively unbalanced dietary structures. Although the proportion of highland barley was declining, and the consumption frequency of highland barley and rice was similar, highland barley was still one of the main staple foods in Tibet. There are both individual and regional differences in dietary and food consumption patterns. Individual factors such as age, education, family member composition, and regional factors, namely time from the township to the county, cultivation area of highland barley, and meat production, might have a considerable influence on Tibetan food consumption. The potential impact of changing the staple food consumption structure and the rationalization of dietary and food consumption patterns for people in remote and environmentally vulnerable areas needs further research attention.

## Figures and Tables

**Table 1:** Food groups and standard weights used in analyses.

Food Items	Food Groups	Weight
Highland barely, rice, wheat, corn, potato, and its products	Main staples	2
Bean and its products	Pulses	3
Vegetables	Vegetables	1
Fruit	Fruit	1
Meat, poultry, and eggs	Meat	4
Milk and its products	Milk	4
Oil	Oil	0.5

**Table 3:** Descriptive statistics of demographic variables (*n* = 506).

Demographic Variable	*n* (Percentage)		
Gender	Male	Female	
	230 (45.45)	276 (54.55)	
Age	<40	40–60	>60
	140 (27.67)	254 (50.20)	112 (22.13)
Educational attainment	No education	Primary	Secondary or higher
	255 (50.40)	189 (37.35)	62 (12.25)
Number of family members	1–5	6–10	11–15
	271 (53.56)	202 (39.92)	33 (6.52)
Number of family members under 18 years old	0–1	2–3	4–6
	225 (44.46)	214 (42.30)	67 (13.24)
Number of family members aged 18–40	0–2	3–5	6–9
	317 (62.65)	176 (34.78)	13 (2.57)
Number of family members aged 40–60	0–1	2–3	4–5
	239 (47.23)	244 (48.22)	23 (4.55)
Number of family members over 60 years old	0	1	2–3
	441 (87.15)	39 (7.71)	26 (5.14)
Percentage of family members under 18 years old	0–30	30.01–60	60.01–100
	258 (50.99)	233 (46.05)	15 (2.96)
Percentage of family members aged 18–40	0–30	30.01–60	60.01–100
	153 (30.24)	301 (59.48)	52 (10.28)
Percentage of family members aged 40–60	0–30	30.01–60	60.01–100
	296 (58.50)	165 (32.61)	45 (8.89)
Percentage of family members over 60 years old	0–30	30.01–60	60.01–100
	486 (96.05)	7 (1.38)	13 (2.57)

**Table 4:** Hierarchical linear model of influencing factors related to food consumption score.

Independent variables	Model 1			Model 2		
	Coefficient	(95% Confidence Interval)		Coefficient	(95% Confidence Interval)	
Constant term	72.85 *	66.29	79.41	74.61 *	66.52	82.71
Age	−0.10	−0.21	0.00	−0.11 *	−0.22	−0.01
No education	Ref			Ref		
Primary	1.66	−1.11	4.44	1.74	−1.02	4.50
Secondary or higher	4.99 *	0.80	9.17	4.69*	0.52	8.86
Number of family members under 18 years of age	−0.13	−1.06	0.81	−0.09	−1.02	0.83
Number of family members aged 18–40 years	0.21	−0.73	1.16	0.26	−0.68	1.21
Number of family members aged 40–60 years	1.74 *	0.54	2.94	1.83 *	0.63	3.03
Number of family members over 60 years of age	0.67	−1.72	3.05	0.81	−1.56	3.17
County of agriculture				Ref		
County of half agriculture and half animal husbandry				1.90	−3.37	7.18
County of animal husbandry				−1.53	−8.83	5.76
The travel time from township to county(minute)				−0.02 *	−0.03	0.00
Cultivation area of highland barley(hectare)				−1.70 × 10^−3^ *	0.00	0.00
Pork, beef, and mutton production(ton)				7.17 × 10^−4^ *	0.00	0.00

Notes: Ref, reference category; *, significant at the 0.05 level.

## Data Availability

The data presented in this study are available on request from the corresponding author and with permission of the Tibet Center of Disease Control and Prevention. The data are not publicly available due to confidentiality requirements.
